# Optimization of CBCT‐guided imaging schedules for left‐sided postmastectomy radiotherapy using VMAT: A dosimetric, radiobiological, and cost analysis

**DOI:** 10.1002/acm2.70629

**Published:** 2026-05-23

**Authors:** Denghong Liu, Changxu Lu, Xueyi Zou, Liping Liu, Lu Liu, Li Zhou

**Affiliations:** ^1^ Radiotherapy Physics & Technology Center, Cancer Center West China Hospital Sichuan University Chengdu P.R. China

**Keywords:** breast cancer, cost analysis, dosimetry, image‐guided schedules, imaging dose, radiobiology

## Abstract

**Purpose:**

This study aimed to determine an appropriate frequency of image‐guided (IG) schedules for left‐sided postmastectomy radiotherapy by comprehensively evaluating dosimetric, radiobiological, imaging dose, and imaging‐related cost outcomes.

**Methods:**

A retrospective analysis of 20 patients treated with volumetric modulated arc therapy (VMAT) was conducted. Virtual CT images were generated by deforming the pCT to daily cone‐beam computed tomography (CBCT) using deformable image registration. Accumulated dose, tumor control probability (TCP), normal tissue complication probability (NTCP), imaging dose, and imaging‐related cost were compared among six IG schedules: no image‐guided (NIG), weekly image‐guided (WIG), twice‐weekly image‐guided (TIG), thrice‐weekly image‐guided (THRIG), initial 3 days then weekly image‐guided (3D + WIG), and daily image‐guided (DIG).

**Results:**

Increasing the frequency of IG schedules significantly improved planning target volume (PTV) dose coverage (D_95_) and TCP (P < 0.001), with DIG achieving optimal target dose delivery. Although no statistically significant differences were observed in dose to organs at risk (OARs) or NTCP, higher‐frequency schedules showed a trend toward reduced variability. However, cumulative imaging dose increased linearly with the frequency of IG schedules (e.g., contralateral breast: 1.26–31.50 mGy; ipsilateral lung: 2.85–71.25 mGy), and imaging‐related cost increased substantially from NIG to DIG, with differences of approximately 1.7‐ to 25‐fold. THRIG demonstrated a favorable balance, with minimal deviations in PTV D_95_ (1.38%) and TCP (0.83%) relative to DIG, while reducing imaging dose and cost to approximately 60% of DIG.

**Conclusion:**

While high frequency IG schedules improve target dose coverage, they are associated with increased imaging dose and imaging‐related cost. Within the CBCT‐guided VMAT workflow evaluated in this study, THRIG may provide a balanced trade‐off between treatment precision, safety, and cost.

## 1 INTRODUCTION

Image‐guided radiation therapy (IGRT) has become a standard procedure for pre‐treatment positional verification in modern radiotherapy.^1^, ^2^ Its primary advantages are twofold: first, by acquiring images immediately before treatment, IGRT enables direct quantification and correction of setup errors, thereby reducing inaccuracies in dose delivery to the target volume and organs at risk (OARs).^3^, ^4^, ^5^ Second, IGRT provides essential technical support for adaptive radiation therapy. Through deformable image registration (DIR) between cone‐beam CT (CBCT) and planning CT (pCT) images, a “virtual CT” reflecting the patient's real‐time anatomy can be generated, allowing for dynamic reassessment of target‐to‐OARs spatial relationships and subsequent plan adjustments.^6^, ^7^, ^8^, ^9^

IGRT, while critical for precise treatment delivery, introduces potential risks primarily associated with additional radiation exposure to patients. Frequent IG schedules may increase the patient's lifetime risk of developing secondary malignancies.^10^, ^11^, ^12^ Ding et al.^13^ demonstrated that imaging dose from a single CBCT scan typically ranges from 3 to 23 cGy, varying with scanning protocol (e.g., half‐fan or full‐fan mode) and treatment site. They further noted that, under a daily CBCT schedule, the cumulative nontherapeutic dose delivered to sensitive organs may approach 300 cGy. This imaging dose is influenced by multiple parameters, including tube voltage (kV), tube current (mA), scan arc, field of view (FOV), and notably, the frequency of IG schedules.^14^, ^15^, ^16^, ^17^ Among these, the frequency of IG schedules represents a modifiable clinical decision and serves as a primary determinant of the imaging dose.^18^ Therefore, the rational optimization of IG schedules presents a significant opportunity to minimize extraneous radiation exposure without compromising target accuracy, forming the central focus of this study.

In addition to balancing precision and safety, imaging‐related cost significantly influences the formulation of clinical IG schedules.^19^, ^20^ For treatment sites with typically minimal setup errors (e.g., the head and neck), the marginal accuracy improvement afforded by high frequency IG schedules may not justify the associated radiation risks and elevated imaging‐related cost.^21^ Conversely, for sites exhibiting pronounced setup variability or where target volume is in close proximity to OARs (e.g., thoracic or abdominal tumors), high frequency IG schedules may prove cost‐effective by decreasing the normal tissue complication probability (NTCP), thereby reducing cost related to managing treatment‐associated toxicity.^22^, ^23^ Therefore, optimizing the frequency of IG schedules is not a uniform decision. Instead, it necessitates striking a dynamic balance among treatment accuracy, cumulative imaging dose, and cost analysis to ensure clinical rationality and practical feasibility.

Currently, the optimal frequency of IG schedules remains a subject of ongoing debate in clinical practice, and no universally accepted standard has yet been established.^24^, ^25^ This lack of consensus has led to persistent variations and inconsistencies in clinical practice. The core of the controversy lies in the need to balance the pursuit of higher treatment precision against the imperative of ensuring radiation safety. A national survey by the American Society for Radiation Oncology (ASTRO) revealed that daily image‐guided (DIG) is employed across most disease sites; however, breast cancer radiotherapy represents an exception.^26^ Different research teams have proposed varying perspectives on IG schedules due to differences in immobilization methods and treatment regimens. For instance, Yao et al.^27^ advocated for DIG in left‐sided whole‐breast irradiation to ensure target dose coverage and reduce dose to OARs. Conversely, Borm et al.^25^ concluded that DIG is unnecessary during breast cancer radiotherapy with a simultaneous integrated boost (SIB) technique.

Given the conspicuous lack of consensus and the critical need to balance precision, safety, and cost, this study aims to conduct a systematic, multifactorial evaluation of various IG schedules. We aim to integrate dosimetric, radiobiological, cumulative imaging dose, and cost analysis to formulate evidence‐based recommendations for optimizing IG schedules in clinical practice for left‐sided postmastectomy radiotherapy.

## 2 MATERIALS AND METHODS

### 2.1 Patient information, immobilization, and CT simulation

A retrospective analysis was conducted on a cohort of 20 breast cancer patients who received left‐sided postmastectomy radiotherapy delivered by an Elekta Synergy linear accelerator (Elekta, Stockholm, Sweden) at our institution between April and August 2025. Patient characteristics are summarized in Table 1. The study was approved by the Institutional Ethics Committee of West China School of Medicine, Sichuan University (Approval No. 2025227).

**TABLE 1:** Patient characteristics.

Characteristics	No. (%)
Cases	20
Age (years)	
Median (range)	55 (27–73)
Weight (kg)	
Median (range)	52 (42–70)
Stage	II‐III
Technology	VMAT
Beam arrangement (roughly)	280°–160°

Abbreviations: VMAT **=** Volumetric modulated arc therapy.

CT simulation was performed using a GE Revolution CT scanner (GE Healthcare, Chicago, USA), with the scanning field covering from the chin to the inferior border of the liver. All images were reconstructed with a slice thickness of 3 mm and subsequently imported into RayStation 10B treatment planning system (RaySearch Laboratories, Stockholm, Sweden) for contouring of the target volume and OARs.

### 2.2 Treatment planning and structure definition

Radiation oncologists contoured the clinical target volume (CTV) and OARs on the pCT in compliance with the guidelines for target delineation and treatment planning of adjuvant radiotherapy for breast cancer.^28^ The planning target volume (PTV) was generated by applying a uniform 5 mm three‐dimensional expansion to the CTV. The contoured OARs specifically included the heart, ipsilateral lung, and contralateral breast. All treatment plans were generated using 6 MV photon beams. The prescribed dose to the PTV was 50 Gy, delivered in 25 fractions, with dose calculation performed using the collapsed cone convolution algorithm. A dual‐arc VMAT technique was employed (gantry angles 280°–160°), ensuring adequate target coverage while sparing OARs.

### 2.3 Generation of virtual CT based on CBCT

For each radiotherapy fraction, initial patient positioning was performed using a laser‐based alignment system by aligning the room's laser lines with skin‐surface fiducial markers. Subsequently, CBCT images were acquired with an Elekta X‐Ray Volumetric Imager (XVI) system under the following parameters: 120 kV tube voltage, S20 collimator, F1 filter, and a total exposure of 146.4 mAs. Gray‐based registration was performed between the acquired CBCT images and the pCT, with the registration focused on the chest wall and supraclavicular lymph nodes. Translational setup errors identified through registration were corrected via the treatment couch's automated adjustment system to meet clinical tolerances.

**FIGURE 1:**
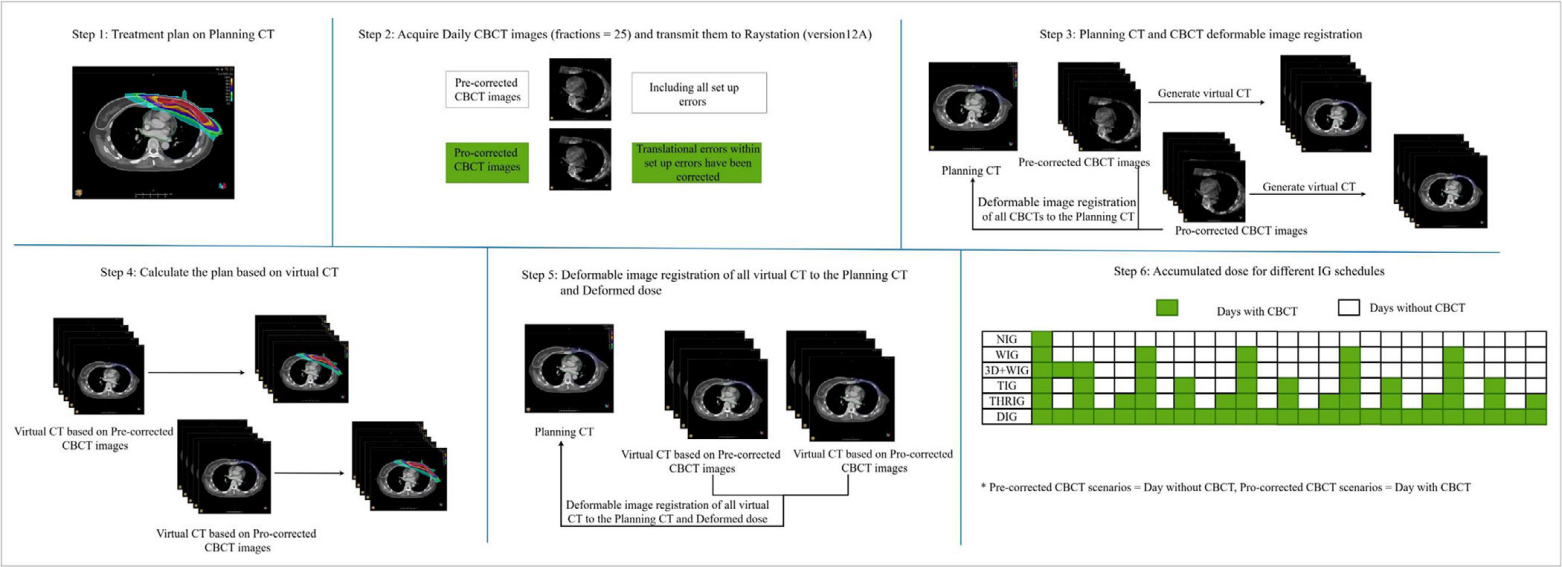
Flowchart for calculating accumulated dose.

Daily CBCT images were reconstructed into pre‐correction and post‐correction images and exported offline to RayStation 12A (RaySearch Laboratories, Stockholm, Sweden). DIR was performed between each CBCT and the pCT, with the pCT designated as the reference image and the daily CBCT as the floating image.^29^, ^30^ The resulting deformation vector fields (DVFs) were used to deform the pCT to the geometry of each CBCT, thereby generating fraction‐specific virtual CT images representing the patient anatomy in both pre‐correction and post‐correction positions. All regions of interest (ROIs), including CTV, PTV, and OARs, were propagated using the same DVFs to ensure spatial consistency between structures and images. The overall workflow is illustrated in Figure 1.

### 2.4 Calculation of accumulated dose using virtual CT

To systematically evaluate the impact of different IG schedules on accumulated dose, the initial treatment plan (including beam angles, dose constraints, and objective weights) was recalculated on each of the previously generated virtual CT images. This yielded the delivered dose distributions for both the pre‐correction and post‐correction positional states of each fraction. Subsequently, to accumulate dose from multiple fractions in the reference frame of the pCT, a second DIR was performed between each virtual CT (for both pre‐ and post‐correction states) and the pCT, with the pCT designated as the reference image and the virtual CT as the floating image. The resulting DVFs were used to deform and map the calculated fractional dose onto the pCT.

Based on the single‐fraction dose distributions obtained above, accumulated dose distributions for the entire treatment course were computed under six IG schedules: no image‐guided (NIG), weekly image‐guided (WIG), twice‐weekly image‐guided (TIG), thrice‐weekly image‐guided (THRIG), initial 3 days then weekly image‐guided (3D+WIG), and daily image‐guided (DIG). For each fraction, if IG was scheduled, the post‐correction dose distribution was used for accumulation; otherwise, the pre‐correction dose distribution was applied. It should be noted that, regardless of the assigned IG schedules, all patients underwent a CBCT scan during the first fraction to verify positioning accuracy and ensure treatment safety.

### 2.5 Calculation of TCP/NTCP under six IG schedules

Tumor control probability (TCP) and NTCP were computed using the linear‐quadratic Poisson model and the Lyman–Kutcher–Burman model, respectively. All radiobiological analyses were performed using Python (version 3.10) with the pyradiobiology package. The required physical dose distributions for each treatment fraction, initially computed by the RayStation 12A, were exported and used as input data for radiobiological modeling. The radiobiological parameters and clinical endpoints utilized for TCP/NTCP computations were consistent with those established in previous studies^31^, ^32^ and are summarized in Table 2.

**TABLE 2:** A parameter set for TCP and NTCP.

ROI	TCD_50_ (Gy)	TD_50_ (Gy)	γ	α/β	m	n	Notices
PTV	39.3		1.7	4			Derivation of a tumor control model from a multi‐center cohort receiving adjuvant radiotherapy.
Heart		48		3	0.1	0.35	Endpoints:pericarditis
Lung		30.8		3	0.18	0.87	Ednpoints:Pneumonitis

### 2.6 Calculation of cumulative imaging dose and imaging‐related cost

The cumulative imaging dose was calculated based on precomputed, Monte Carlo‐simulated dose data specific to the Elekta XVI system (as reported by Marchant et al.^33^), using the scanning parameters detailed in Table 3. For each image‐guided schedule, the cumulative dose (*D*_cum_) was derived by multiplying the simulated dose from a single CBCT scan (*D*_single_) by the frequency (*F*) performed under that schedule, as expressed in the following Equation (1):

(1) $$D_{cum}=D_{single}\times F$$

**TABLE 3:** Imaging dose and imaging‐related cost.

Metrics	No. (%)
Scanning Parameters	Preset
kV	120
Collimator	S20
Filter	F1
Total mAs	117.1
Rotation	half
Organ imaging dose (mGy)	
Lung	2.85
Contralateral breast	1.26
Imaging‐related cost (CNY)	
Single cost	288

Similarly, the total direct imaging‐related cost for each IG schedule was evaluated in accordance with the institutional medical service fee schedule. The total direct imaging‐related cost (*C*_total_) was calculated as the product of the single direct imaging‐related cost (*C*_single_) and the frequency (*F*) performed under that schedule, according to Equation (2):

(2) $$C_{total}=C_{single}\times F$$

### 2.7 Statistical analysis

Statistical analyses were performed using Python with the SciPy, pandas, and NumPy packages. Normality of data distributions was assessed using the Shapiro–Wilk test. Overall differences among groups were evaluated using the Friedman test. Pairwise comparisons between groups were conducted using paired *t*‐tests with Bonferroni correction for multiple comparisons. Statistical significance was set at *P* < 0.05.

## 3 RESULTS

### 3.1 Dosimetric comparison of six IG schedules

**FIGURE 2:**
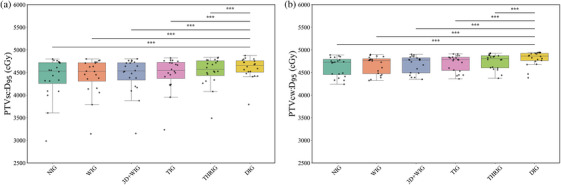
Dosimetric comparison of PTV: D_95_ among six image‐guided schedules. Notes: Statistical significance levels are denoted as follows: **P < 0.05; ***P < 0.001.

As shown in Figure 2, significant differences in D_95_ were observed for both the supraclavicular (PTVsc, Panel a) and chest wall (PTVcw, Panel b) among six IG schedules when compared to DIG (*P* < 0.001). The NIG schedule resulted in the lowest D_95_ for both target volumes. With increasing frequency of IG schedules, a progressive improvement in D_95_ was observed, culminating in the highest values under the DIG schedule. Under DIG, median D_95_ values were highest and closest to the prescribed dose, confirming that DIG provides optimal target dose coverage for PTVsc and PTVcw.

**FIGURE 3:**
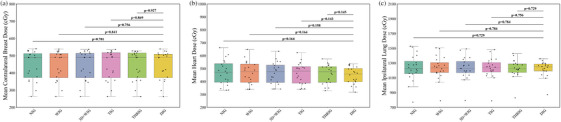
Dosimetric comparison of mean dose to OARs among six image‐guided schedules. Notes: **P < 0.05; ***P < 0.001.

Figure 3 illustrates that the mean dose to the contralateral breast, heart, and ipsilateral lung showed no statistically significant differences among the evaluated IG schedules (*P* > 0.05). Analysis of median values and interquartile ranges further revealed that as the frequency of IG schedules increased, the mean dose to OARs became more concentrated—exhibiting reduced variability—while consistently maintaining low dose levels.

### 3.2 TCP/NTCP comparison among six IG schedules

**FIGURE 4:**
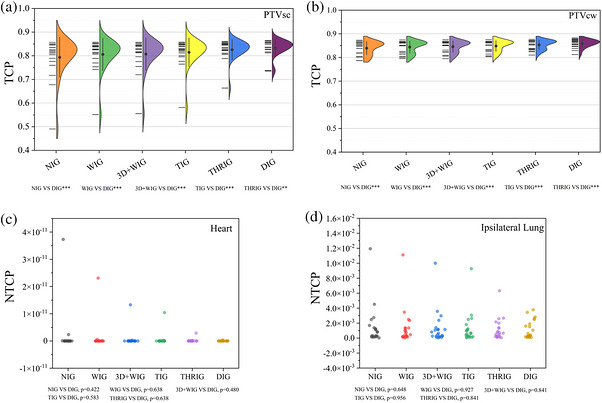
Radiobiological comparison of TCP and NTCP among six image‐guided schedules. Notes: **P < 0.05; ***P < 0.001.

Figure 4 presents the impact of six IG schedules on TCP and NTCP. A significant increase in TCP was observed for both PTVsc and PTVcw as the frequency of IG schedules increased (P < 0.001). Under the NIG schedule, PTVsc exhibited a broad TCP distribution, with a minimum value of 0.49. As the frequency of IG schedules increased, TCP values became more concentrated toward higher levels, peaking under the DIG schedule. Although PTVcw followed a similar trend, its TCP values were consistently superior to those of PTVsc among all schedules, indicating greater robustness to reductions in the frequency of IG schedules.

No statistically significant differences in NTCP were detected for the heart or ipsilateral lung among IG schedules (P > 0.05). The heart NTCP remained negligible under all conditions. With increasing frequency of IG schedules, the ipsilateral lung NTCP remained at low levels.

### 3.3 Imaging dose and imaging‐related cost comparison among six IG schedules

**FIGURE 5:**
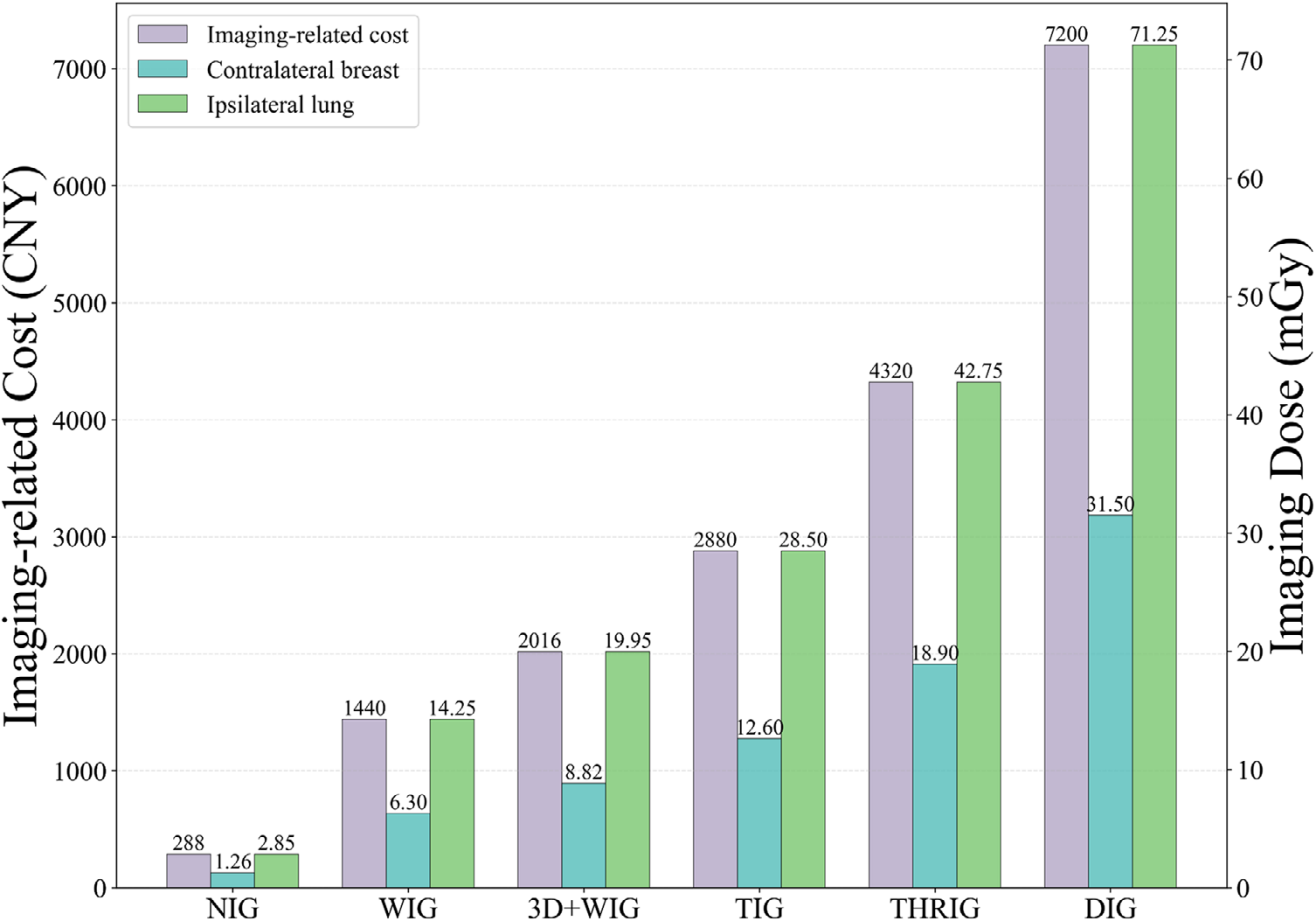
Imaging dose and imaging‐related cost analysis of six image‐guided schedules.

Figure 5 illustrates the proportional relationship between frequency and both cumulative imaging dose and total imaging‐related cost. As the frequency of imaging increased, the contralateral breast dose rose from 1.26 mGy under the NIG schedule to 31.50 mGy under the DIG schedule, while the ipsilateral lung dose increased from 2.85 mGy to 71.25 mGy. A similar trend was observed for total imaging‐related cost, with the DIG schedule incurring the highest expenses, approximately 1.7‐ to 25‐fold higher than those of the other IG schedules.

### 3.4 Comparative analysis of alternative schedules versus the DIG schedule

**FIGURE 6:**
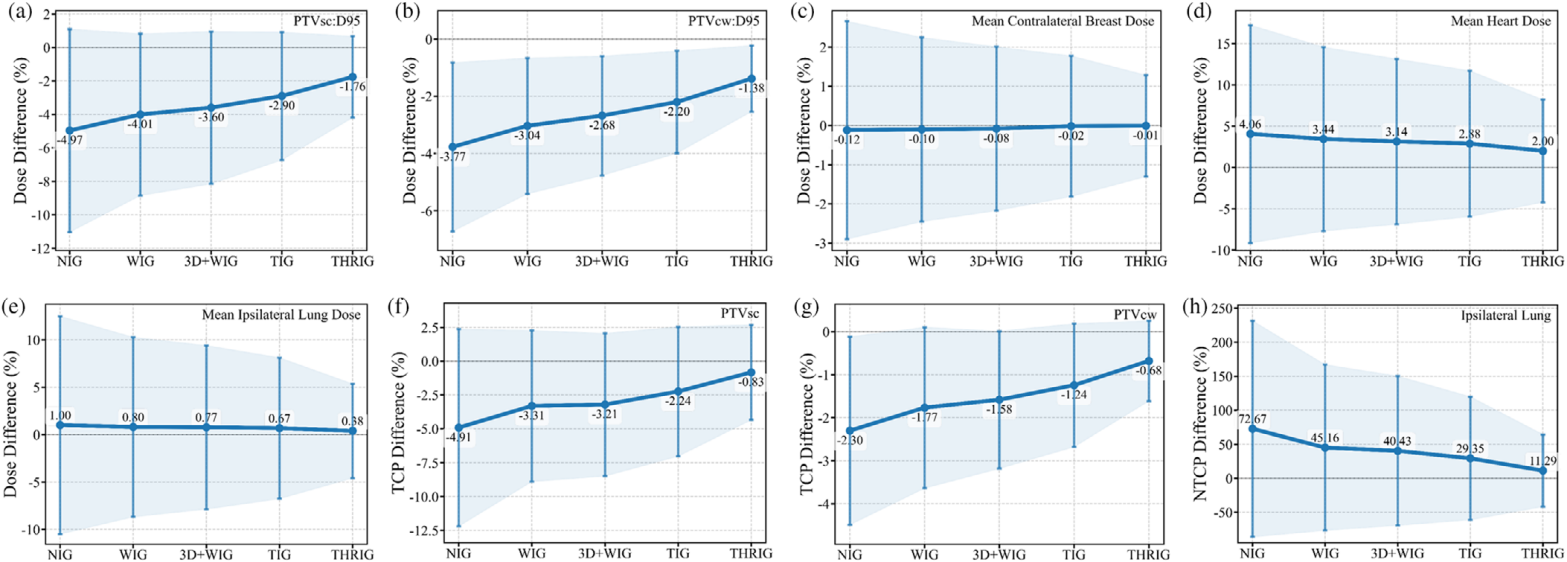
Comparative analysis of alternative schedules versus the DIG schedule.

Figure 6a–e compare the dose differences in target volume and OARs among various IG schedules relative to DIG. The dose differences of PTVcw were consistently smaller than those of PTVsc. Among OARs, the heart exhibited the largest dose differences, followed by the ipsilateral lung, whereas the contralateral breast exhibited minimal differences. Notably, among intermediate‐frequency schedules, THRIG demonstrated the smallest dose deviations in both target volumes and OARs, suggesting its potential to maintain dosimetric accuracy while preserving OARs sparing.

From a radiobiological perspective (Figure 6f–h), the TCP differences of PTVcw were also consistent with the dosimetric findings, exhibiting smaller differences compared to that of PTVsc. Ipsilateral lung NTCP exhibited a relatively substantial difference. Heart NTCP values were clinically negligible among all schedules and thus excluded from analysis. THRIG again exhibited minimal differences from DIG in both TCP and NTCP.

## 4 DISCUSSION

The determination of an appropriate frequency of IG schedules remains a topic of ongoing debate in clinical practice, and no universally accepted standard has been established. Previous studies have predominantly focused on comparing the necessity of DIG with low‐frequency schedules such as TIG, thereby leaving the comprehensive assessment of a wider range of IG frequencies largely unexplored.^15^, ^25^ To address this gap, we evaluated six IG schedules using dosimetric, radiobiological, imaging dose, and cost analysis.

Dosimetric analysis revealed a significant upward trend in PTV dose coverage with increasing frequency of IG schedules. Among these schedules, DIG exhibited the highest D_95_ for both PTVsc and PTVcw, most closely approximating the prescribed dose and confirming its role in achieving optimal dosimetric precision.^4^, ^27^, ^34^ Notably, PTVsc exhibited a greater dose discrepancy (3.28%) than PTVcw (2.61%) when the frequency of IG schedules was reduced. This finding is consistent with Wang et al.’s residual error analysis,^30^ which highlighted PTVsc susceptibility to dose variations. Radiobiological analysis further supported these results: DIG produced TCP significantly higher than other schedules, with values for PTVcw consistently exceeding those of PTVsc, reflecting its greater intrinsic stability. These findings highlight the need for IG schedules tailored to the specific anatomical and geometric characteristics of each target volume, as also suggested by Yao et al.^27^ Specifically, their study recommended DIG for left‐sided breast cancer and proposed WIG as a potential optimal choice for right‐sided breast cancer.

Analysis of OARs showed that mean doses to the heart, ipsilateral lung, and contralateral breast were not significantly affected by the frequency of IG schedules. The heart's NTCP remained negligible among all schedules, whereas a modest but observable increase in ipsilateral lung NTCP was associated with high frequency IG schedules. Greater dose differences were observed in OARs among different IG schedules compared with those observed in the target volume. This may be attributed to the matching focus adopted in this study, which prioritized the chest wall and supraclavicular regions. Zhou et al.^35^ reported that different matching focuses resulted in variations in setup errors. Therefore, the results of the present study may vary depending on the selected matching focus.

While high frequency IG schedules offer distinct advantages in dosimetric precision, their associated drawbacks, including increased imaging dose and substantial imaging‐related cost, require careful consideration. As expected, cumulative imaging dose and imaging‐related cost increased proportionally with the frequency of IG schedules. These findings provide a quantitative basis for evaluating the trade‐offs among treatment efficacy, patient safety, and cost considerations when selecting an IG schedule. Further supporting the risk assessment, our previous research quantified the lifetime attributable risk (LAR) of secondary malignancies associated with cumulative imaging dose, reporting estimated incidences per 100 000 persons of 78 for brain cancer, 271 for lung cancer, and 510 for leukemia based on organ‐specific exposures.^36^ Beyond concerns regarding the LAR of secondary malignancies, the cost implications of high frequency IG schedules are considerable. Lee et al.^37^ reported a 1.5‐fold increase in radiotherapy cost in South Korea between 2016 and 2020, largely driven by technological advancements. This may increase patients’ financial burden and limit adoption in resource‐limited settings.

In summary, clinical decisions regarding IG schedules should comprehensively consider dosimetry, radiobiology, imaging dose, and cost. The present study demonstrates that THRIG may represent a balanced option within the evaluated workflow. The differences in PTV D_95_ and TCP between THRIG and DIG were minimal, at merely 1.38% and 0.83%, respectively, and remained within the ± 5% dose homogeneity range recommended in ICRU Report 83.^38^ In contrast, the cumulative imaging dose and imaging‐related cost associated with THRIG were reduced to only 60% of those observed with DIG. It should be noted that this analysis considered only direct imaging‐related costs and did not include indirect costs such as staff time or equipment depreciation. Furthermore, surface‐guided radiotherapy (SGRT) presents a viable, radiation‐free supplemental modality for patient positioning on days when CBCT is not scheduled.^39^, ^40^ Peng et al.^41^ proposed distinct “CBCT + SGRT” modes, suggesting that daily SGRT combined with CBCT at varying frequencies can dynamically monitor tumor positional variations while reducing additional radiation exposure.

Several limitations of this study warrant consideration. First, the accuracy of virtual CT generated from CBCT is inherently limited by image quality and potential residual registration errors, which may introduce uncertainty in dose recalculation.^30^, ^42^ However, all IG schedules were evaluated under consistent conditions, supporting the validity of comparative analyses. Second, the cumulative imaging dose was estimated using Monte Carlo‐simulated data for the Elekta XVI system rather than patient‐specific measurements. Third, this study was conducted within a CBCT‐guided VMAT workflow at our institution. However, treatment technique (e.g., IMRT or VMAT) may affect sensitivity to setup errors and consequently influence the optimal IG schedules.^43^ These findings are specific to the CBCT‐guided VMAT workflow and may vary with alternative techniques and clinical settings. Finally, the cost analysis included only direct imaging‐related cost based on institutional fee schedules, without accounting for indirect costs, revenue cycle factors, or regional variability, which may limit broader applicability. Future research will focus on prospective clinical validation and development of individualized predictive models to optimize IG schedules. This model will integrate and quantify the relative contributions of key determinants, including dosimetric accuracy, radiobiological parameters, cumulative imaging dose, and cost considerations, while being tailored to individual patient characteristics (e.g., probability of setup error occurrence, treatment technique, and financial capacity), to enable dynamic optimization of IG schedules.

## 5 CONCLUSION

Within a CBCT‐guided VMAT workflow, high frequency IG schedules improve PTV dose coverage and TCP without significant changes in OAR dose or NTCP, but increase imaging dose and cost. Therefore, IG schedules should be carefully selected by balancing accuracy, safety, and cost. In our institutional setting, THRIG achieved comparable target coverage to the daily IG schedule with reduced imaging dose and lower cost, and may represent a practical compromise.

## AUTHOR CONTRIBUTIONS

Denghong Liu designed the study, collected data, analyzed data, and drafted the manuscript. Changxu Lu, Xueyi Zou, Liping Liu, and Lu Liu collected data. Li Zhou designed the study, revised and finally approved the manuscript. All authors read and confirmed the manuscript.

## CONFLICT OF INTEREST STATEMENT

The authors have no relevant conflicts of interest to disclose.

## ETHICS STATEMENT

The study was approved by the Institutional Ethics Committee of West China School of Medicine, Sichuan University (Approval No. 2025227).

## Data Availability

The datasets used and/or analyzed during the current study are available from the corresponding author on reasonable request.
